# Supplementary material for: Safety and Efficacy of Incorporating Actellic® 300 CS into Soil Wall Plaster for Control of Malaria Vectors in Rural Northeastern Uganda
Source: Trop Med Infect Dis. 2024 Dec 24;10(1):4. doi: 10.3390/tropicalmed10010004 (PMC11768474; doi:10.3390/tropicalmed10010004)
Supplement: Supplementary file 1 [file tropicalmed-10-00004-s001.zip › tropicalmed-3209120-supplementary.pdf]

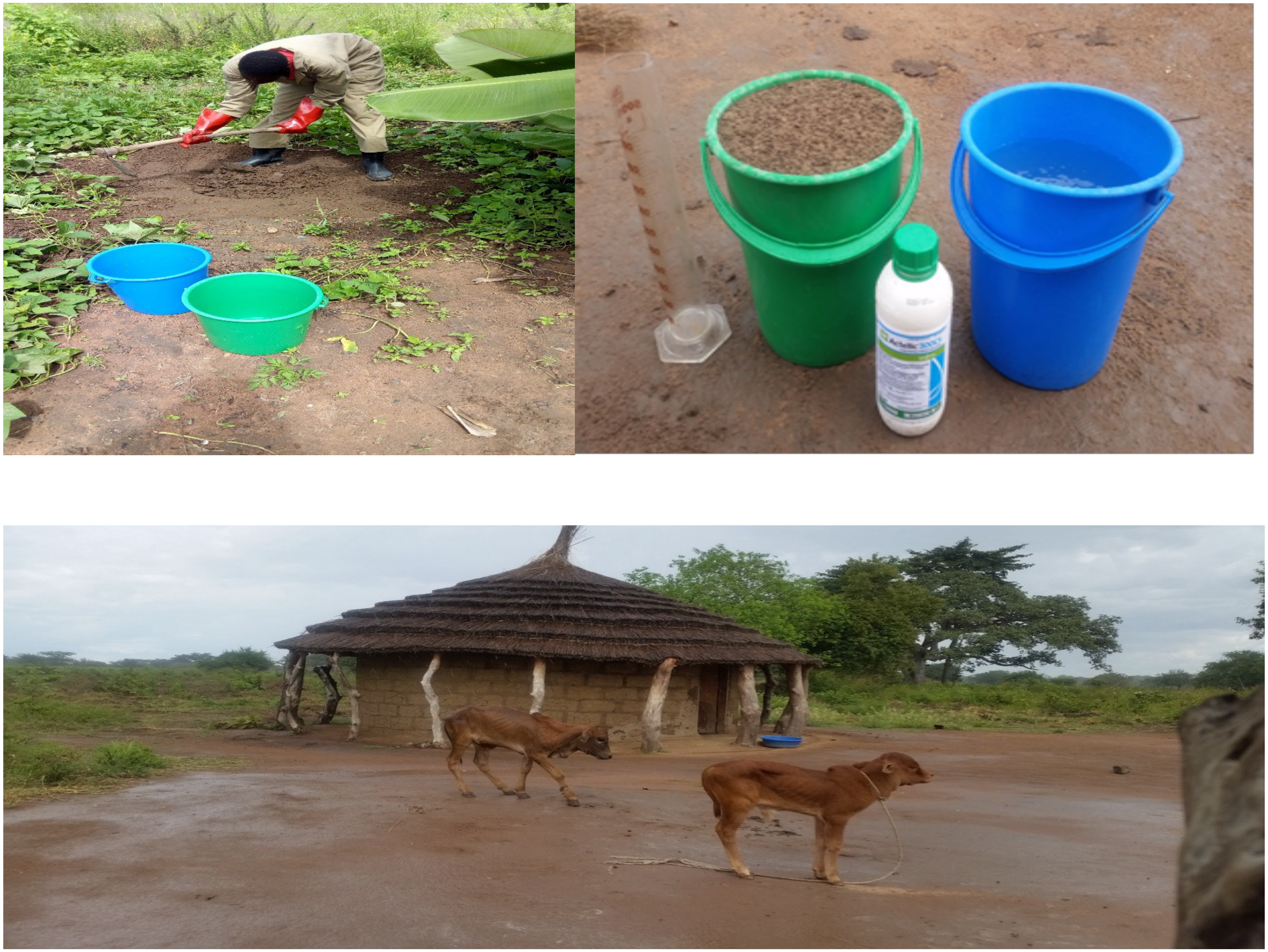

Figure 1: Top Left: Digging up soil from the homestead garden. Top Right: Components of HD4MC: soil, water, and insecticide; Bottom left: A typical circular hut

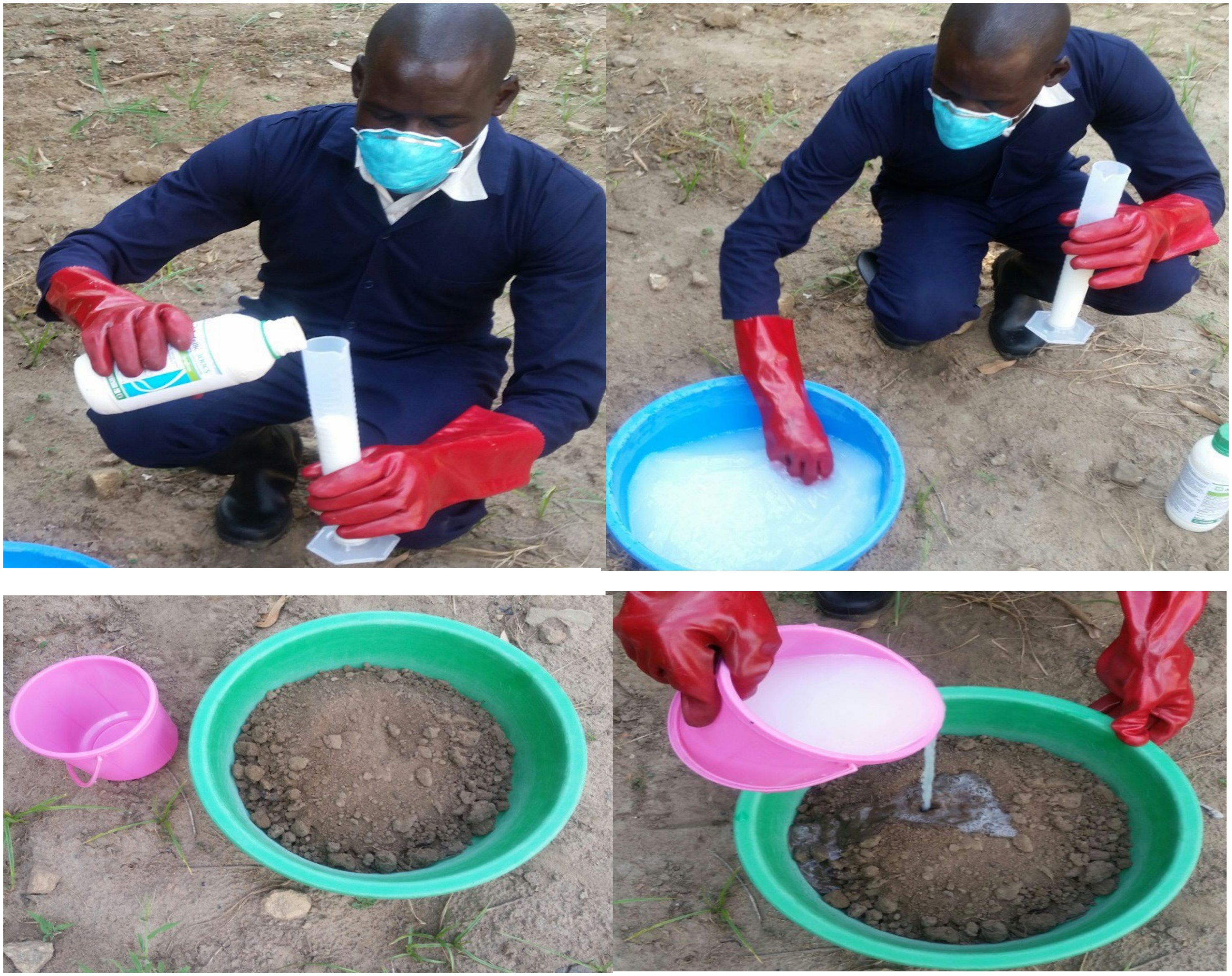

Figure 2: Top left: Measuring the calculated volume of insecticide. Top right: Mixing insecticide with water. Bottom left: Soil in basin measured in 2-liter buckets. Bottom right: adding insecticide/water mix to soil

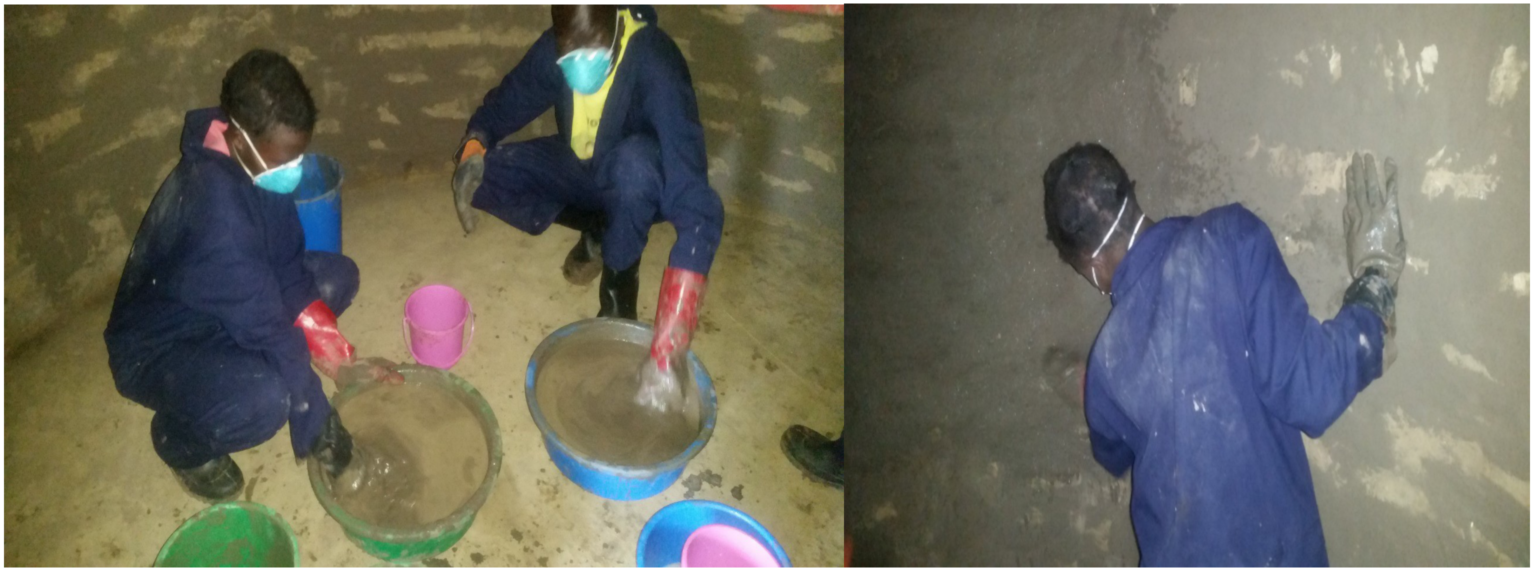

Figure 3: Left: Preparing plaster mix of the correct consistency. Right: Spreading the plaster

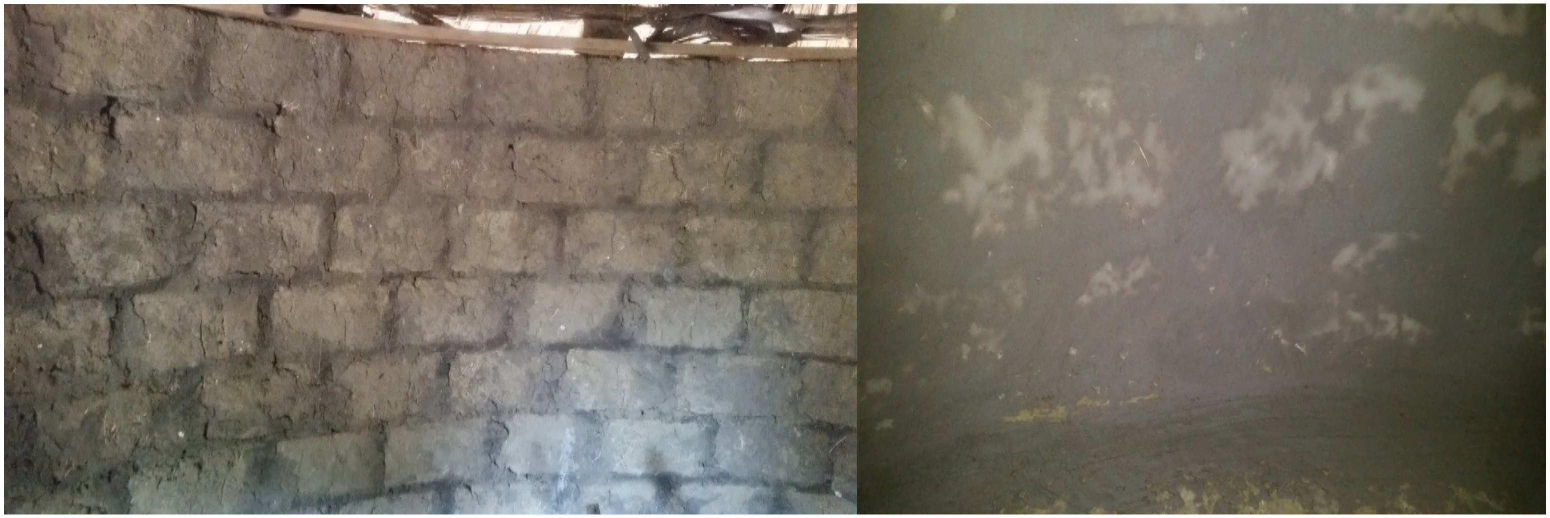

Figure 4: Left: wall surface BEFORE smearing. Right: wall surface AFTER smearing. Notice the smooth finish with no 'bubbles' or flakes of plaster.

Supplemental Table 1: Baseline malaria vector feeding capacity

Ongema					Otujai					Acurun									Abwokodia								
	An. gambiae s.l.					An. gambiae s.l.					An. gambiae s.l.				**An. funestus**					An. gambiae s.l.				**An. funestus**			
HH No (Pop)	**UF**	**F**	**HG**	**G**	HH No, (Pop)	**UF**	**F**	**HG**	**G**	HH No, (Pop)	**UF**	**F**	**HG**	**G**	**UF**	**F**	**HG**	**G**	HH No, (Pop)	**UF**	**F**	**HG**	**G**	**UF**	**F**	**HG**	**G**
1 (5)	0	1	1	1	1 (1)	0	0	0	0	1 (2)	0	0	4	2	0	0	3	0	1 (1)	0	0	2	1	0	0	0	0
2 (2)	0	4	3	4	2 (2)	0	3	3	1	2 (7)	0	2	1	0	0	2	12	0	2 (3)	0	0	0	1	0	0	0	0
3 (2)	0	2	5	2	3 (5)	0	0	1	0	3 (2)	1	1	0	1	0	0	4	0	3 (2)	0	2	0	0	0	0	0	0
4 (4)	0	2	2	2	4 (3)	0	0	1	1	4 (2)	0	0	0	1	0	1	0	0	4 (4)	0	1	1	1	0	0	0	0
5 (4)	0	3	7	13	5 (6)	0	3	7	3	5 (3)	1	0	0	0	0	4	5	0	5 (2)	0	0	2	1	0	0	2	0
6 (3)	0	0	1	0	6 (3)	0	0	0	2	6 (1)	1	0	0	1	0	0	0	0	6 (4)	0	1	0	0	0	0	0	0
7 (1)	0	1	1	1	7 (2)	0	0	0	0	7 (1)	1	0	0	0	0	0	0	0	7 (5)	0	0	0	1	0	0	0	0
8 (4)	0	7	7	1	8 (2)	0	0	1	1	8 (3)	0	0	3	0	0	0	8	2	8 (2)	0	0	1	0	0	0	1	0
9 (2)	0	1	1	0	9 (1)	0	0	0	0	9 (3)	1	1	0	1	0	2	1	1	9 (1)	0	0	0	0	0	0	0	0
10 (6)	0	0	1	0	10 (2)	0	0	1	0	10 (2)	1	0	0	0	0	0	0	0	10 (2)	0	0	0	0	0	0	0	0
11 (4)	0	0	0	0	11 (1)	0	0	0	1	11 (4)	0	0	2	1	0	3	8	0	11 (1)	0	0	0	1	0	0	0	0
12 (1)	0	1	1	0	12 (5)	0	1	1	3	12 (2)	1	0	1	1	2	2	0	0	12 (2)	0	0	0	0	0	0	0	0
**38**	**0**	**22**	**30**	**24**	**33**	**0**	**7**	**15**	**12**	**32**	**7**	**4**	**11**	**8**	**2**	**14**	**41**	**3**	**29**	**0**	**4**	**6**	**6**	**0**	**0**	**3**	**0**

UF= Unfed; F= Fed; HG= Half gravid; G= Gravid; HH No. = household number; Pop = household population

Supplemental Table 2: Feeding pattern of species-specific female Anopheles collected using HLC indoors per hour

Collection Time	***Otujai***		**Abwokodia**		**Acurun**		**Total**	
	An. Gambiae s.l	***An. funestus***	***An. Gambiae s.l***	***An. funestus***	***An. Gambiae s.l***	***An. funestus***	***An. Gambiae s.l***	***An. funestus***
6 – 7 p.m.	0	0	0	0	3	0	3	0
7– 8 p.m.	3	0	0	0	2	2	5	2
8 – 9 p.m.	4	1	0	0	9	0	13	1
9 – 10 p.m.	0	0	3	1	6	2	9	3
10 - 11 p.m.	5	0	2	2	20	6	27	8
11 – 12 a.m.	13	0	10	4	19	7	42	11
12 - 1 a.m.	5	0	18	0	20	15	43	15
1 - 2 a.m.	9	0	8	1	36	21	53	22
2 - 3 a.m.	23	2	9	0	38	27	70	29
3 - 4 a.m.	26	0	6	4	26	27	58	31
4 - 5 a.m.	7	0	6	2	28	23	41	25
5 - 6 a.m.	8	1	9	0	30	19	43	20
**Total**	**103**	**4**	**71**	**14**	**237**	**149**	**411**	**167**
